# An Efficient Approach for Pixel Decomposition to Increase the Spatial Resolution of Land Surface Temperature Images from MODIS Thermal Infrared Band Data

**DOI:** 10.3390/s150100304

**Published:** 2014-12-25

**Authors:** Fei Wang, Zhihao Qin, Wenjuan Li, Caiying Song, Arnon Karnieli, Shuhe Zhao

**Affiliations:** 1 School of Geographic and Oceanographic Sciences, Nanjing University, Nanjing 210023, China; E-Mails: essiwf@163.com (F.W.); 2007songcaiying@163.com (C.S.); 2 Institute of Agro-Resources and Regional Planning, Chinese Academy of Agricultural Sciences, Beijing 100081, China; E-Mail: liwj@caas.net.cn; 3 The Remote Sensing Laboratory, Department of Environmental Physics J. Blaustein Institute for Desert Research, Ben Gurion University of the Negev, Sede Boker Campus, Midreshet Ben-Gurion 84990, Israel; E-Mail: karnieli@bgu.ac.il; 4 Collaborative Innovation Center of South China Sea Studies, Nanjing University, Nanjing 210023, China

**Keywords:** pixel decomposition, land surface temperature, spatial resolution, MODIS, ASTER

## Abstract

Land surface temperature (LST) images retrieved from the thermal infrared (TIR) band data of Moderate Resolution Imaging Spectroradiometer (MODIS) have much lower spatial resolution than the MODIS visible and near-infrared (VNIR) band data. The coarse pixel scale of MODIS LST images (1000 m under nadir) have limited their capability in applying to many studies required high spatial resolution in comparison of the MODIS VNIR band data with pixel scale of 250–500 m. In this paper we intend to develop an efficient approach for pixel decomposition to increase the spatial resolution of MODIS LST image using the VNIR band data as assistance. The unique feature of this approach is to maintain the thermal radiance of parent pixels in the MODIS LST image unchanged after they are decomposed into the sub-pixels in the resulted image. There are two important steps in the decomposition: initial temperature estimation and final temperature determination. Therefore the approach can be termed double-step pixel decomposition (DSPD). Both steps involve a series of procedures to achieve the final result of decomposed LST image, including classification of the surface patterns, establishment of LST change with normalized difference of vegetation index (NDVI) and building index (NDBI), reversion of LST into thermal radiance through Planck equation, and computation of weights for the sub-pixels of the resulted image. Since the Advanced Spaceborne Thermal Emission and Reflection Radiometer (ASTER) with much higher spatial resolution than MODIS data was on-board the same platform (Terra) as MODIS for Earth observation, an experiment had been done in the study to validate the accuracy and efficiency of our approach for pixel decomposition. The ASTER LST image was used as the reference to compare with the decomposed LST image. The result showed that the spatial distribution of the decomposed LST image was very similar to that of the ASTER LST image with a root mean square error (RMSE) of 2.7 K for entire image. Comparison with the evaluation DisTrad (E-DisTrad) and re-sampling methods for pixel decomposition also indicate that our DSPD has the lowest RMSE in all cases, including urban region, water bodies, and natural terrain. The obvious increase in spatial resolution remarkably uplifts the capability of the coarse MODIS LST images in highlighting the details of LST variation. Therefore it can be concluded that, in spite of complicated procedures, the proposed DSPD approach provides an alternative to improve the spatial resolution of MODIS LST image hence expand its applicability to the real world.

## 1. Introduction

MODIS thermal infrared (TIR) band data are mainly used to retrieve land surface temperature with such techniques as split window algorithms for the study of land surface energy budgets [1,2], water resource management [3,4], agricultural drought [5,6], and environmental biogeochemistry processes [7]. However, spatial resolution of the data is 1000 m under nadir, which is very low in comparison with that of visible and near-infrared band data (for example, 250 m for bands 1 and 2). The retrieved LST images from MODIS TIR data are also with much low spatial resolution (1000 m under nadir), which has limited their applications in many studies requiring high spatial resolution to identify detailed variation of thermal heat flux over the region under study [8]. Therefore, it is very necessary to increase the spatial resolution of MODIS LST images in order to meet the needs of many studies [9,10] requiring pixel scale to identify the details of LST variation under complicated environments[11,12].

To increase the spatial resolution of an image actually means to decompose its pixels into smaller ones. A number of studies have been done for visible and infrared band data [13–15]. Data fusion has been a well-known technique to integrate multi-sensors or multi-sources image data with different spatial resolutions (hence pixel scales) into an image with better pixel scale [16,17]. Gao *et al.*, proposed the spatial and temporal adaptive reflectance fusion model (STARFM) for fusing Landsat and MODIS surface reflectance data to produce a synthetic “daily” surface reflectance product at the ETM + spatial resolution [18]. Hilker *et al.*, improved the model through adding the day of disturbed information, extracted from the time-series MODIS images, to reduce the possible errors in the resulting fused image [19].

Several attempts have also been made to increase the spatial and temporal resolutions of thermal infrared imagery or LST data [20–22]. Guo and Moore developed a pixel block intensity modulation (PBIM) method to improve 120 m spatial resolution of Landsat TM thermal band 6 image into 30 m through integrate topographic details in reflective spectral bands for each thermal pixel block. The method improves the thermal variation caused by topography to 30 m resolution while maintaining the average thermal digital number (DN) unchanged [22]. Liu and Pu developed two methods to downscale the coarse resolution TIR radiance for sub-pixel temperature retrieval [23]. By considering the relationship of LST or emissivity between NOAA-AVHRR and Landsat TM data, Stathopoulou and Cartalis improved the PBIM method by defining a scaling factor to downscale AVHRR LST [24]. Nichol developed an emissivity modulation with a simplified assumption that thermal emissivity was uniform within the low-resolution pixels to decompose the images with coarse pixel scale into fine one using the emissivity estimated from the auxiliary data with the same pixel scale as the decomposed one [25]. Recently attempts have been made to use the spectral unmixing model and artificial neural network for disaggregation of the TIR or LST data [20,21].

Due to very high temporal resolution, MODIS data have been widely used for various studies. The low spatial resolution of MODIS thermal band data have been a major obstacle for application of the data to many studies such as urban heat island monitoring and agro-drought monitoring that required high spatial resolution to highlight details of spatial variation in the region under study. Several efforts have been made to down-scale the coarse pixel of MODIS thermal infrared band data into better spatial resolution [11]. The down-scaling was generally done through the well-known LST-NDVI relationship. Kustas *et al.*, developed an approach called DisTrad method to establish a simple linear regression equation between LST and NDVI to decompose MODIS LST image into a better spatial resolution [26]. Essa *et al.*, improved the evaluation DisTrad (E_DisTrad) method for urban areas [27].

NDVI is not only one of the factors affecting variation of LST in the real world [12]. Other relevant indices have also been used for decomposition of MODIS thermal band data [27,28]. An approach using fractional vegetation cover instead of NDVI was developed in Agam *et al.*, for thermal band data sharpening [29]. Stathopoulou and Cartalis used the intensity of surface urban heat island to downscale the AVHRR LST images into the same spatial resolution as Landsat TM 6 band data [24]. Essa *et al.*, compared the applicability of 15 different indices for pixel decomposition of MODIS LST images and found that the correlation between LST and the impervious percentage was the best in urban areas [30]. Zakšek and Oštir decomposed the pixels of MODIS LST images through principal components and regression equation within a moving widow over the assistant image with high resolution [12]. Jing and Cheng used a non-linearly transformation to produce the maximal correlation between MODIS TIR bands and multiple reflective bands for pixel decomposition of MODIS LST images [28]. These attempts of pixel decomposition usually are able to generate a result that is acceptable in image interpretation of thermal infrared data. However, they are not able to maintain the thermal infrared radiance of the pixels unchanged after decomposition.

Objective of this paper is to develop an efficient approach for pixel decomposition of MODIS LST image to increase its spatial resolution while the thermal radiance of sub-pixels in the resulted decomposed LST image remains as the same of the parent pixel from which the sub-pixels are generated. How to maintain the radiance of LST image pixels unchangeable while decomposing them properly is the core breakthrough of this study. Since two steps are involved in our proposed approach for pixel decomposition of MODIS LST image: initial temperature estimation for the sub-pixels in the resulted LST image and final temperature determination through weighting approach to maintain the thermal radiance unchanged after decomposition, we term it the double-step pixel decomposition (DSPD). After describing the theoretical basis and the image processing procedures of the DSPD approach, we designed an experiment to validate it through comparison with evaluation DisTrad (E-DisTrad) method developed by Essa *et al.* [27], which is very close to our DSPD method in the first step of pixel decomposition. Therefore, the paper is organized as a methodology section presenting the details of the DSPD approach and a result section to validate the approach as well as a conclusion to summarize the key points of the study.

## 2. Background

To increase the spatial resolution of a MODIS LST image actually means to decompose its pixels into smaller ones with relative high spectral multispectral image as assistance. Since ASTER was on board the same Terra platform as MODIS for Earth observation, the spatial resolution of MODIS LST can be decomposed from 1000 m into 250 m with its VNIR data or 90 m with ASTER visible/infrared (VNIR) data. The LST observed by ASTER can be the true value to validate the MODIS LST decomposed result, so the LST product and multi-spectral data of MODIS and ASTER are chosen as our experiment data.

### 2.1. Dataset

**Figure 1.**
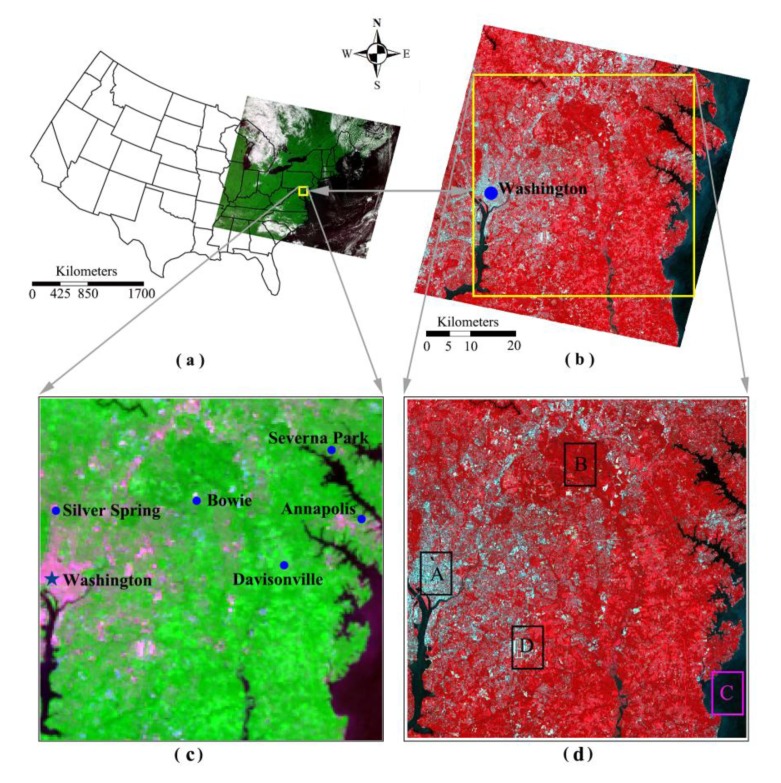
The location of the data used of the approach through experiment with the MODIS and ASTER images on-board the same Terra platform of remote sensing system for Earth observation, (**a**) the MODIS image and its geographical location in the eastern part of USA, RGB: 321; (**b**) the ASTER image, RGB: 3N21; (**c**) the MODIS subset covering the Washington DC and Maryland region; (**d**) the ASTER subset covering the same region. The rectangles A, B, C, and D are the interested areas for comparison.

For the application site of Washington (Figure 1), the MODIS LST product (MOD11A1), MODIS emissivity product (MOD11A1, MOD11B1), ASTER LST product (AST_08), ASTER emissivity product (AST_05) and ASTER multi-spectral data (AST_L1B) are collected. A pair of MODIS and ASTER images were obtained for this experiment. The images were acquired at 16:03 Greenwich time (12:03 US east time) on 24 August 2003. The MODIS image covers the eastern region of USA (Figure 1a), while the ASTER image only occupies a small part of the MODIS image due to its very high spatial resolution. Figure 1b shows the ASTER image. After geometric correction, the pixel scale of the MODIS image is 1000 m while the scale of ASTER image is 90 m.

In order to validate the approach, a small typical subset of image covering the same region was made from the MODIS and ASTER images. Figure 1c shows the subset from MODIS image with 50 × 50 pixels and Figure 1d is the subset from ASTER image with 550 × 550 pixels. The subset covers parts of Washington DC and Maryland region, with Chesapeake Bay in its east. Three surface patterns can be easily identified in the subsets: urban region, water surface and natural terrain.

### 2.2. Pre-Processing of the Dataset

Since the spatial resolution and data format are different between MODIS LST product and VNIR data, the dataset need to do some pre-processing before they can be used. The coordinate reference system of MODIS product need to be converted to the UTM/WGS84 same as the ASTER data. Then an image-to image georeferencing is performed by collecting many dispersed ground control points (GCPs) throughout each image and applying 1st-order polynomial transformation to match the ASTER data with MODIS using the nearest neighbor resampling method. Then the co-registered images are overlaid over one another in order to examine potential pixel misalignement caused by different spatial resolution of MODIS and ASTER. Indeed, some misalignment was visually detected, which is further corrected by the upper-left corner of the ASTER image [24].

## 3. Methodology

### 3.1. Theoretical Principle of DSPD

The TIR band data of remote sensing system such as MODIS are usually with relative much lower spatial resolution than the reflective ones (usually in VNIR spectral region) on the same system due to the fact that TIR data were obtained through detecting the ground-emitted thermal radiance which is much weaker than the ground-reflected solar irradiance which becomes the energy for optical imaging system according to spectral theorem described by Planck equation. As to MODIS, the LST product retrieved from MODIS TIR data (bands 29–36) are with a spatial resolution of 1000 m under nadir viewing while VNIR data (bands 1 and 7) are with the resolution of 250 m for bands 1 and 2 or 500 m for bands 3–7. This coarse pixel scale of the MODIS LST images has limited their application to environmental issues requiring high spatial resolution to detect the exiguous variation of thermal properties.

One way to solve the problem of low spatial resolution of MODIS LST images is to increase their spatial resolution through so-called pixel decomposition techniques. In this paper we intend to develop a new approach for the decomposition of MODIS LST images. Since the existing methods for decomposition of thermal infrared data and its retrieved LST images does not keep the thermal radiance of pixels unchanged after they are decomposed, our goal in the study is to break through this limit to maintain the thermal radiance in the decomposed image equal to their parent pixel from which they are generated.

Decomposition of MODIS LST images usually requires assistance of auxiliary data. In our case, the available auxiliary data covering the same region as the LST image are the MODIS VNIR band data such as bands 1 and 2 with pixel scale of 250 m or bands 3–7 in 500 m. Therefore, our specific goal is to develop an approach for pixel decomposition to increase the 1000 m spatial resolution of MODIS LST images into the same scale of 250 m using the available MODIS VNIR band data (e.g., bands 1 and 2) as auxiliary data.

**Figure 2.**
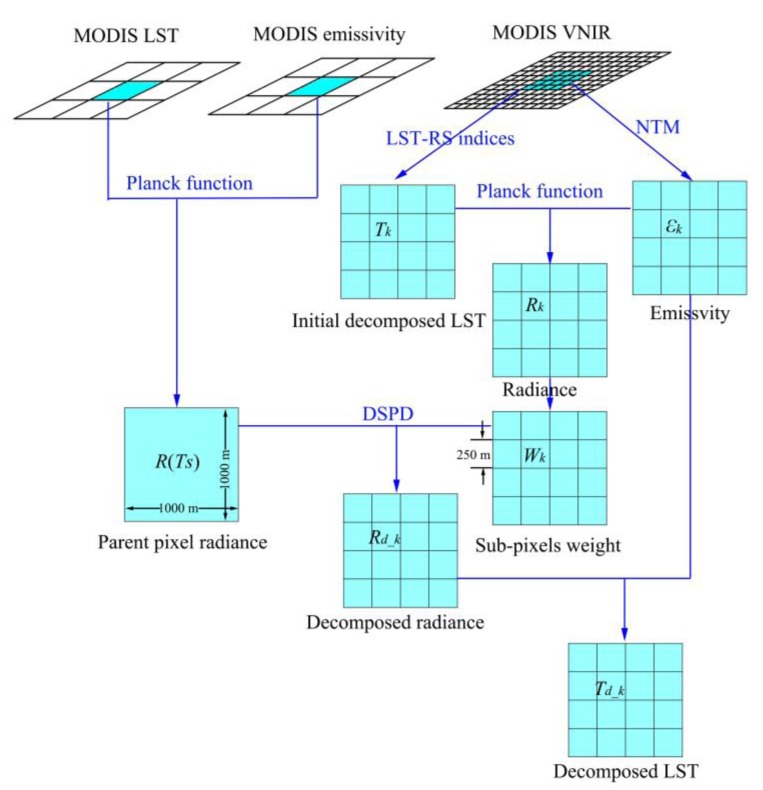
Theoretical principle of our DSPD approach for pixel decomposition of MODIS LST image with its VNIR/SWIR data.

Figure 2 illustrates the main theoretical principle of our approach for pixel decomposition. In order to divide the 1000 m MODIS LST image pixel into the decomposed sub-pixels of 250 m scale and to maintain the thermal radiance of the parent pixel unchanged, we have to use the auxiliary data to compute a weight for each of the decomposed sub-pixels so that the total thermal radiance of the parent pixel can be distributed among the sub-pixels. The final result of decomposed LST image can be computed using the relationship between thermal radiance and surface temperature for each sub-pixel.

Therefore, for a parent pixel *i* in the MODIS LST image (Figure 2), we can use the known land surface temperature (*T_s_*) to compute its thermal radiance *R*(*T_s_*) as follows using the Planck equation formula about the radiance at a given wavelength range [31–33]:

(1) $$R\left(T_{s}\right)={ɛ}_{i}R_{b}\left(T_{s}\right)={ɛ}_{i}\int_{\lambda_{1}}^{\lambda_{2}}\frac{C_{1}}{\lambda^{5}\left(e^{C_{2}/\lambda T_{s}}-1\right)}$$

where *R*(*T_s_*) is the thermal radiance with land surface temperature *T_s_* at given wavelength range (W·m^−2^) of the parent pixel *i* in MODIS LST image; ε*_i_* is the emissivity of the pixel at given wavelength range λ_1_–λ_2_ (μm); *R_b_*(*T_s_*) (Unit, W·m^−2^) is the blackbody thermal radiance at a given wavelength range λ_1_–λ_2_ (μm). *T_s_* is the land surface temperature (K); *C*_1_ and *C*_2_ are the radiance constants with *C*_1_ = 1.191 × 10^8^ W·μm^4^·sr^−1^·m^−2^ and *C*_2_ = 1.439 × 10^4^ μm K respectively.

For convenient calculation and model, the relationship between thermal radiance of blackbody (*R_b_*) and temperature (*T_s_*) in specific wavelength (e.g., 8–13.5 μm, 10.78–11.28 μm) range will be found:

(2) $$R_{b}\left(T_{s}\right)=\frac{K_{1}}{\exp\left(K_{2}/T_{s}\right)-1}$$

where *K*_1_ and *K*_2_ are the coefficient of thermal radiance and temperature of the blackbody at different wavelength range given in Table 1.

If we are able to find a radiance weight for each sub-pixel in the decomposed LST image, the thermal radiance of the sub-pixels can be easily computed as follows:

(3) $$R_{d\_k}=W_{k}\;R(T_{s})\;\text{for}k=1,2,3.\ldots 16$$

where *R_d_k_* is the thermal radiance of sub-pixel *k*, and *W_k_* is the weight of the sub-pixel *k, R*(*T*_s_) is the thermal radiance of the parent pixel. Since the pixel scale of auxiliary data (e.g., MODIS NVIR bands 1 and 2) is 250 m, the number of the decomposed sub-pixels for the parent pixel should be 16. In order to maintain the thermal radiance of the parent pixel i unchanged, we have:

(4) $$\sum_{k=1}^{16}W_{k}/16=1$$

Then the LST of each sub-pixel in the decomposed LST image can then be computed with Equations (1) and (2) as follows:

(5) $$T_{d\_k}=\frac{K_{2}}{\ln\left(1+{ɛ}_{k}K_{1}/R_{d\_k}\right)}$$

where *T_d_k_* is the decomposed LST for the sub-pixel *k* in the decomposed LST image.

In order to use Equation (5) to compute the land surface temperature for the decomposed pixels, one has to determine the weight and the emissivity for each sub-pixel. This can be done through the auxiliary VNIR band data.

### 3.2. Determination of Weight for the Pixel Decomposition

The radiance weight is needed to the computation of thermal radiance for each sub-pixel in the decomposed LST image. Then the LST of each sub-pixel could be calculated by its decomposed thermal radiance. Since we are not able to directly obtain the true decomposed temperature for determination of the weight to decompose the parent pixel, we use other estimated temperature for computation of weight for the sub-pixels and we term the estimated temperature at this stage the initial temperature for the sub-pixels.

If we can estimate the initial decomposed temperature (*T_k_*) and emissivity (ε*_k_*) for each sub-pixel of the decomposed LST image as shown in Figure 2, we are able to compute the thermal radiance at given wavelength of each sub-pixel as follows using Planck equation formula about the radiance as follows:

(6) $$R_{k}={ɛ}_{k}R_{b}\left(T_{k}\right)=\frac{{ɛ}_{k}K_{1}}{\exp\left(K_{2}/T_{k}\right)-1}$$

where *R_k_* is the estimated thermal radiance for sub-pixel *k, T_k_* and ε*_k_* are the estimated initial temperature and emissivity of the sub-pixel. Therefore the total thermal radiance of the sub-pixels can be estimated as follows:

(7) $$R_{\text{tot}}=\sum_{k=1}^{16}R_{k}/16$$

where *R_tot_* is the total thermal radiance of the decomposed sub-pixels from the parent pixel. In order to meet the Equation (4) to maintain the thermal radiance of the parent pixel unchanged after decomposed, the weight for each sub-pixel can be determined as follows:

(8) $$W_{k}=R_{k}/R_{tot}=16R_{k}/\sum_{k=1}^{16}R_{k}$$

Therefore the importance for weight determination is to properly estimate the emissivity and the initial temperature for each sub-pixel *k*.

### 3.3. Estimation of Emissivity for the Sub-Pixels

It is well known that emissivity of an object is mainly determined by its thermo-physical characteristics of the object. For the ground surface, the components composing of the surface are the main factors determining the ground emissivity [32,34,35]. Many effective methods have been approved to obtain the emissivity, e.g., the Day/Night method [36], grey body emissivity method [37], *et al.*, Since the emissivity at different spatial resolution is needed in the decomposition process, we should find a method to estimate the emissivity of MODIS at different resolution. So the NDVI thresholds method (NTM) [38] is chosen to estimate the emissivity respectively at resolution of 250 m and 1000 m.

While the emissivity is variable with the wavelength, the NTM can estimate the emissivity of different land surface accurately at 10–12 μm range, so we choose the wavelength of band 31 (10.78–11.28 μm) for emissivity estimation and thermal radiance calculate based on the MODIS band wavelength design. At this wavelength range, the emissivity can be modeled as follows [38]:

(9) $${ɛ}_{\lambda }=\{\begin{array}{ll} {ɛ}_{s\lambda }, & \text{NDVI}<{\text{NDVI}}_{s}\\ {ɛ}_{v\lambda }P_{v}+{ɛ}_{s\lambda }\left(1-P_{v}\right)+C_{\lambda }, & {\text{NDVI}}_{s}\leq \text{NDVI}\leq {\text{NDVI}}_{v}\\ {ɛ}_{v\lambda }P_{v}+C_{\lambda }, & \text{NDVI}>{\text{NDVI}}_{v} \end{array}$$

Subject to:

(10) $$C_{\lambda }=\left(1-{ɛ}_{s\lambda }\right){ɛ}_{v\lambda }F^{\prime }\left(1-P_{v}\right)$$

where ε_λ_ is the band emissivity, ε*_v_*_λ_ and ε*_s_*_λ_ are respectively the vegetation and soil emissivity, P*_v_* is the proportion of vegetation, *C* is a term due to surface roughness (*C* = 0 for flat surface), NDVI*_v_* and NDVI*_s_* are the NDVI for a fully vegetated pixel and soil one respectively, *F′* is a geometrical factor ranging between zero and one.

Usually vegetation cover fraction at pixel scale can be computed from its NDVI as follows [39,40]:

(11) $$P_{v}={\left[\frac{\text{NDVI}-{\text{NDVI}}_{s}}{{\text{NDVI}}_{v}-{\text{NDVI}}_{s}}\right]}^{2}$$

Over particular areas, NDVI*v* and NDVI*s* values can be extracted from the NDVI histogram. Values of NDVI*_v_* = 0.5 and NDVI*_s_* = 0.2 were proposed by in to apply the method in global conditions [38]. While the value for vegetated surfaces (NDVI*_v_* = 0.5) may be too low in some cases, for higher resolution data over agricultural sites, the NDVI*_v_* can reach 0.8 or 0.9 [41].

### 3.4. Estimation of the Initial Temperature for the Sub-Pixel

Since the core of our approach for pixel decomposition is to divide the LST of the parent pixel into the decomposed sub-pixels, the temperature of the sub-pixel is the final result of the decomposition that we intend to obtain from the decomposition. Thus, we are not able to use the true decomposed temperature for determination of the weight to decompose the parent pixel. We have to use other approaches to estimate the temperature for computation of weight for the sub-pixels and we term the estimated temperature at this stage the initial temperature for the sub-pixels.

**Figure 3.**
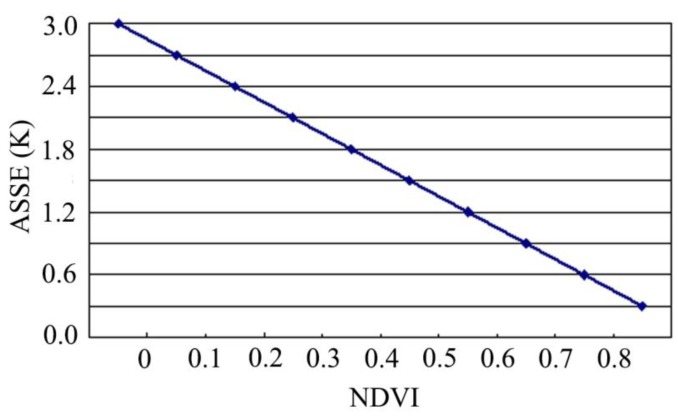
The ASEE as a function of NDVI for estimation of initial temperature to determine the weight for pixel decomposition of MODIS LST image.

It has been well known that land surface temperature was determined by the surface structure shaping the thermal performance of the ground surface [26,42,43]. Vegetation has been recognized as the most important factor governing the performance of LST variation at regional scale [29]. Thus the relationship between vegetation index and LST has been extensively examined for various applications to such issues as drought and evaporation [4,5], urban heat island [20] and spatial sharpening of thermal imagery [29]. In the study we also use the relationship between NDVI and LST to estimate the initial temperature of the sub-pixels for weight determination. Among the three patterns of ground surface, natural terrain has the most obvious feature of vegetation dynamics. Thus the relationship between NDVI and LST is mainly used to estimate the initial temperature for the sub-pixels categorized as the pattern of natural terrain. The establishment is done over the MODIS LST image and the re-sampled NDVI image retrieved from the auxiliary VNIR band data to match the pixel scale of LST image at 1000 m. Therefore we have the relationship for the initial temperature estimation as follows:

(12) $$T_{ns}=a_{n}+b_{n}{\text{NDVI}}_{r}$$

where *T_ns_* is the LST for the pixels of natural terrain in the LST image, *NDVI_nr_* is the vegetation index in the resampled NDVI image of the pixels, *a_n_* and *b_n_* are the regression coefficients between LST and the NDVI. This regression is usually with a standard error of estimation (SEE) due to the fact that the relationship is not actually with a rigorous linearity but with a vibration on both sides. Therefore we improved the relationship by consideration of this estimate error and then applied to the retrieved NDVI image for the estimation of initial temperature of the sub-pixels as follows:

(13) $$T_{nk}=a_{n}+b_{n}{\text{NDVI}}_{s}+R_{n}\left(T\right)$$

where *T_nk_* is the estimated initial temperature of the sub-pixels classified as natural terrain pattern; *NDVI_ns_* is the NDVI of the sub-pixels computed from the auxiliary VNIR band data; *a_n_* and *b_n_* are regression coefficients obtained from Equation (18), *R_n_*(*T*) is a function to generate random number using computer clock as the initial for randomization. Since regression of the relationship between LST and NDVI is generally with a SEE, the inclusion of the random number by the function is conFigured to be as follows:

(14) $$R_{n}\left(T\right)\leq \pm {\text{ASEE}}_{n}$$

where *ASEE_n_* is the adjusted SEE, which should be as a function of NDVI with a maximum of ±3 K according to our experiences. This is because the vibration of LST for the same NDVI level is with a trend of increasing for a decreasing of NDVI in magnitude. Therefore, for sub-pixels with high value of NDVI, the LST is generally low and with a vibration to be also small. As a contrast, the LST is usually not only very high but also with high vibration for the pixels with a low value of NDVI (Figure 3). Accordingly we have the function of *ASEE_n_* to NDVI for the natural terrain pattern as follows:

(15) $${\text{ASEE}}_{n}=\{\begin{array}{lll} 3 & \text{for} & \text{NDVI}<0.1\\ 3-0.3\text{NDVI} & \text{for} & 0.1\leq \text{NDVI}\leq 0.8\\ 0.3 & \text{for} & \text{NDVI}>0.8 \end{array}$$

Though natural terrain occupies most of the ground in pixel scale, urban region and water are also the common surface patterns that we encounter in remote sensing images. This is especially true when high spatial resolution images are under studies. For the VNIR band data with pixel scale of 250 m, the two surface patterns also frequently appear. To estimate the initial temperature for the two patterns, we follow the same methodology as done for natural terrain. Instead of NDVI, the normalized difference of building index (NDBI) and water color index (WCI) are used for the estimation of initial temperature, respectively as follows:

(16) $$T_{mk}=a_{m}+b_{m}{\text{NDBI}}_{s}+R_{m}\left(T\right)$$

(17) $$T_{wk}=a_{w}+b_{w}{\text{WCI}}_{s}+R_{w}\left(T\right)$$

where *T_mk_* and *T_wk_* are the estimated initial temperature for the sub-pixels of building surface and water bodies, *NDBI_s_* and *WCI_s_* are the normalized difference of building index and water color index for the two surface patterns respectively, *a_m_* and *b_m_* are the regression coefficients between LST and NDBI, *a_w_* and *b_w_* are the regression coefficients between LST and WCI, *R_m_* (*T*) and *R_w_* (*T*) are the random function for the two surface patterns, which can be given according to the magnitude of their surface temperature vibration as follows:

(18) $$R_{m}\;\left(T\right)\leq \pm 4.0$$

(19) $$R_{w}\;\left(T\right)\leq \pm 0.3$$

Thus we have the initial temperature determination as follows:

(20) $$T_{k}=\{\begin{array}{ll} T_{nk} & \text{for natural terrain}\\ T_{mk} & \text{for urban region}\\ T_{wk} & \text{for water body} \end{array}$$

where *T_k_* is the estimated initial temperature for weight determination in our approach.

### 3.5. Estimation of the Important Parameters for the Approach

Three important parameters are required to determine for the approach: NDVI, NDBI and WCI. Since we use MODIS bands 1 and 2 as our auxiliary data for the decomposition of LST image, the parameter NDVI for the auxiliary data can be computed as follows:

(21) $$\text{NDVI}=\frac{B_{2}-B_{1}}{B_{2}+B_{1}}$$

where *B*_2_ and *B*_1_ are the pixel value of MODIS bands 2 and 1 respectively. Atmospheric correction should be done to the two band data before they are used to compute the NDVI.

The normalized difference of building index (NDBI) and water color index (WCI) can be computed as follows:

(22) $$\text{NDBI}=\frac{B_{6}-B_{2}}{B_{6}+B_{2}}$$

(23) $$\text{WCI}=\{\begin{array}{ll} 1 & \text{for DN}2>4000\\ DN2/4000 & \text{for}\;0\leq DN1\leq 4000 \end{array}$$

where *B*_6_ is the pixel value of MODIS band 6, and ND2 is the DN value of water pixels in MODIS band 2. Therefore, the value of WCI ranges from 0–1.0. The color of water tends to be light as WCI increases.

### 3.6. Procedures of the Approach for Pixel Decomposition

The procedures to conduct pixel decomposition with the approach can be summarized as follows:

(1) Preparing the required data. This includes the MODIS LST and emissivity product with pixel scale of 1000 m for the pixel decomposition and the required auxiliary data which is the MODIS VNIR band data (e.g., bands 1 and 2) with pixel scale of 250 m, and band 6 with pixel scale of 500 m. Resampling MODIS band 6 to pixel scale of 250 m matching that of bands 1 and 2. Geometric correction is required to be done for the LST image and the VNIR band data so that they are coordinately matched with each other to cover the same geographical region.
(2) Classifying the auxiliary data. A classified image with three surface patterns for the pixels is generated: natural terrain, urban region, and water body.
(3) Estimating the essential parameters. This is to compute the four essential parameters (e.g., NDVI, NDBI, WCI, and Pv) from the auxiliary data, resulting in four corresponding parameter images with pixel scale of 250 m. The parameter images are then re-sampled to the pixel scale of 1000 m.
(4) Estimating emissivity. This is done to the auxiliary NDVI data (250 m) with the NTM, resulting in an emissivity image at the wavelength of 10.78–11.28 μm required to compute the weight.
(5) Establishing regressions equations for the 3 surface patterns. This includes to take a samples of pixels from the MODIS LST image and the re-sampled image of the corresponding parameter at pixel scale of 1000 m and then to carry on regression analysis between LST and the corresponding parameters to determine the coefficients of the regression equations.
(6) Estimating the initial temperature. This is to apply the regression equations obtained in step 5 to the auxiliary data with pixel scale of 250 m, using the classified image and the parameter images as assistance.
(7) Determining the weight. This includes to compute the thermal radiance from the initial temperature image and the emissivity image with pixel scale of 250 m and to summarize thermal radiance for each block with 4 × 4 pixels in order to match the pixel scale of the MODIS LST image.
(8) Computing thermal radiance of the sub-pixels with scale of 250 m. This includes to compute the thermal radiance of the LST image for each parent pixel and to compute the thermal radiance for each of the sub-pixels in the block.
(9) Reversing temperature from the thermal radiance of the sub-pixels to generate the final result of decomposing the LST image.

**Figure 4.**
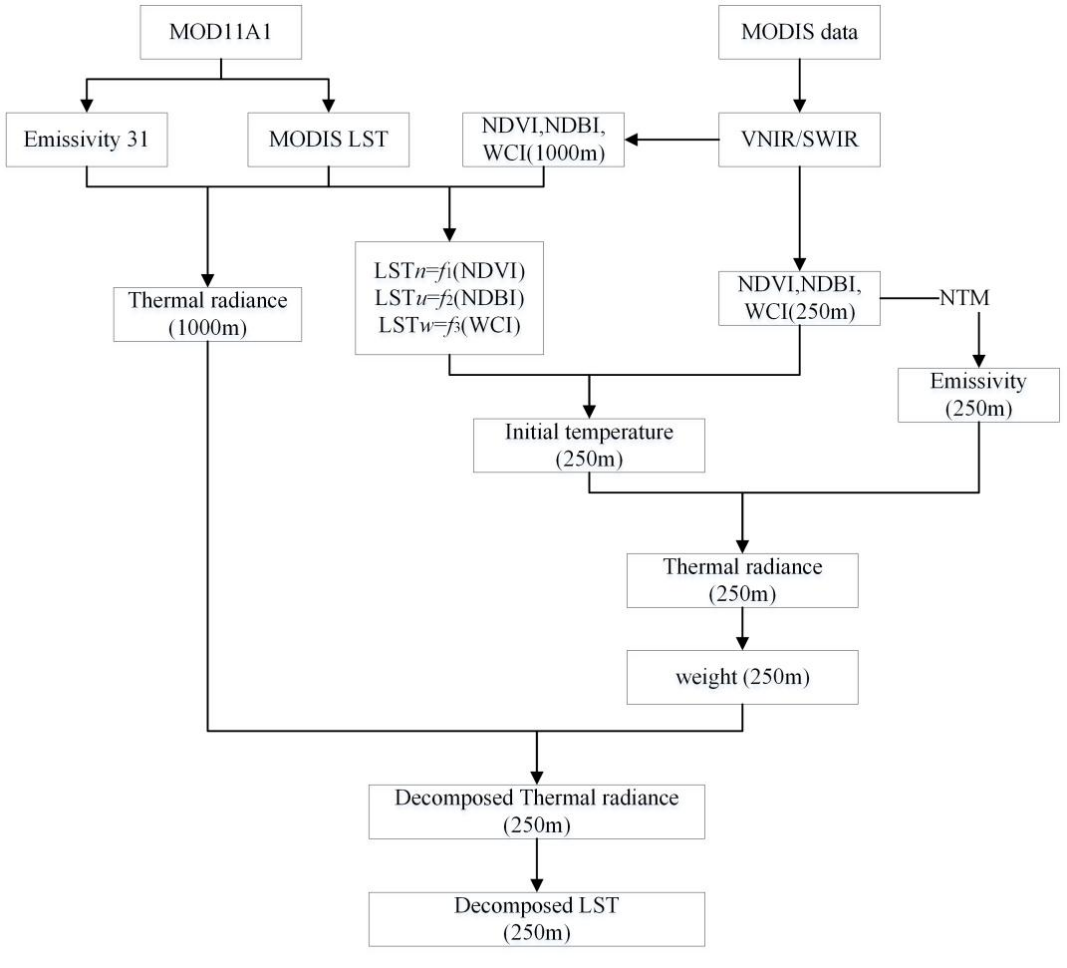
Technical process of the DSPD for pixel decomposition of MODIS LST image with its VNIR/SWIR data.

The above procedures are illustrated in Figure 4 showing the technical process of the approach.

### 3.7. Experiments for Validation of the Approach

The best way to validate the applicability of the approach is to compare the decomposed LST image with the simultaneous measurement of LST in the same geographical region. Since ASTER was on board the same Terra platform as MODIS for Earth observation, we can use ASTER LST product to validate the decomposed LST image if we can find a pair of ASTER and MODIS images matching precisely the acquisition time and place. We can use the ASTER LST product to validate the decomposed LST image if we can find a pair of ASTER and MODIS images matching precisely the acquisition time and place. Spatial resolution of ASTER LST data is with pixel scale of 90 m. Therefore we can decompose the MODIS LST image into the same pixel scale as ASTER LST image to check the accuracy of the approach, which can be assessed through the root mean error, standard deviation and mean square error between the ASTER LST and the decomposed LST image:

(24) $$ME=\sum \left(LST_{m}-LST_{a}\right)/N$$

(25) $$\text{STD}=\sqrt{{\sum \left(LST_{m}-LST_{a}-ME\right)}^{2}/N}$$

(26) $$\text{RMSE}=\sqrt{\sum {\left(LST_{m}-LST_{a}\right)}^{2}/N}$$

where *ME, STD* and *RMSE* are respectively the mean error, standard deviation and root mean square error of the decomposed LST image from the MODIS LST image in comparison with the ASTER LST image, *LST_m_* and *LST_a_* are the decomposed LST and the ASTER LST respectively for the corresponding pixels in scale of 90 m. *N* is the pixel number of the ASTER LST image.

**Figure 5.**
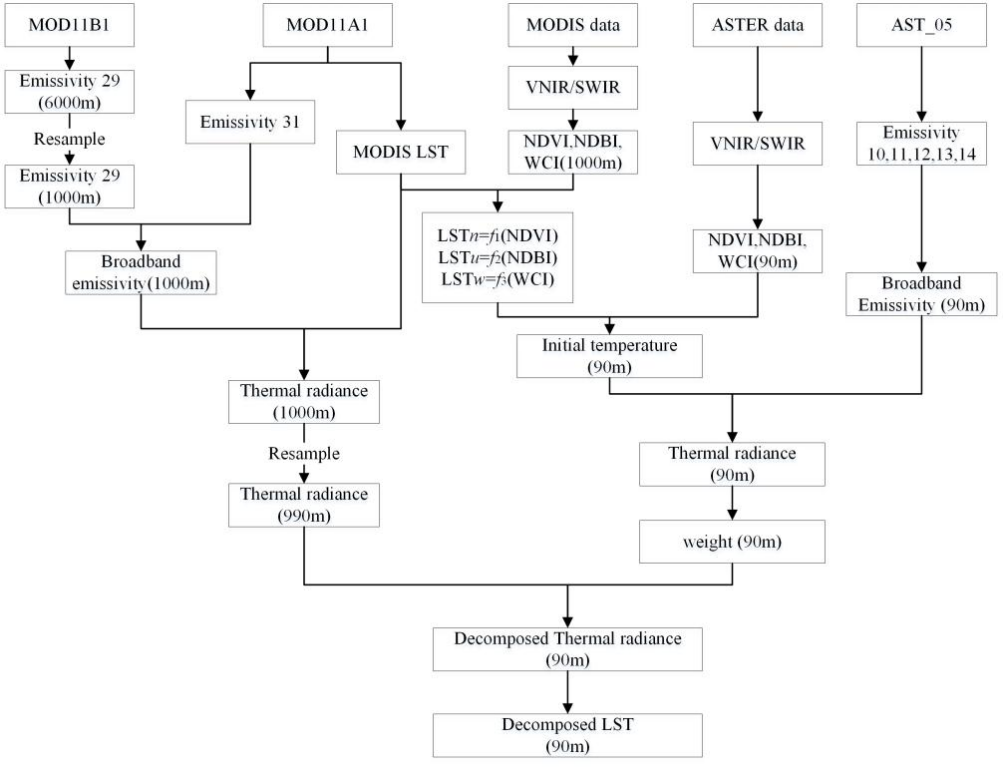
Technical process of the DSPD for pixel decomposition of MODIS LST image with ASTER VNIR/SWIR data.

In order to conduct the validation, a series of procedures have to be conducted for computation of LST error statistics indices in Equations (24)–(26). As shown in the Figure 5, the decomposition process has some difference with the MODIS decomposition with its VNIR. The difference is mainly focus on the thermal radiance calculation with ASTER and MODIS. Because the wavelength range of the MODIS and ASTER thermal bands are different, and the effective emissivity varies with different wavelength range. The radiance cannot be compared directly between MODIS and ASTER. So the broadband emissivity (BBE) is used to calculate the thermal radiance of MODIS and ASTER in wavelength range 8–13.5 μm. Ogwa *et al.*, showed that surface broadband emissivity could be estimated as a linear combination of the narrowband emissivity estimates in the range of 8–13.5 μm [33,44]. And Cheng *et al.*, give the broadband emissivity estimation method of ASTER and MODIS with their narrow thermal band emissivity [31]:

(27) $${ɛ}_{bb\_ast}=0.197+0.025{ɛ}_{10}+0.057{ɛ}_{11}+0.237{ɛ}_{12}+0.333{ɛ}_{13}+0.146{ɛ}_{14}$$

(28) $${ɛ}_{bb\_\text{mod}}=0.095+0.329{ɛ}_{29}+0.572{ɛ}_{31}$$

where ε*_bb_ast_* and ε*_bb_mod_* are respectively the BBE of MODIS and ASTER, ε_10_ to ε_14_ are the ASTER thermal narrowband emissivity from band 10 to 14. ε_29_ and ε_31_ are the MODIS thermal narrowband emissivity for bands 29 and 31.

Similarly the thermal radiance of ASTER and MODIS pixel at 8–13.5 μm could be calculated as follow:

(29) $$R_{\text{mod}}\left(T\right)=\frac{{ɛ}_{bb}K_{1}}{\exp\left(K_{2}/T\right)-1}$$

Then procedures to conduct MODIS LST pixel (1000 m) decomposition into ASTER LST (90 m) with the approach can be summarized as follows:

(1) MODIS thermal radiance calculation: we will calculate the broadband emissivity with the MODIS emissivity of band 29 and 31. Then the MODIS thermal radiance at the wavelength range 8–13.5 μm will be calculate by the broadband emissivity and MODIS LST. Re-sampling the MODIS thermal radiance to 990 m with the nearest neighbor method.
(2) Re-sampling the ASTER VNIR (15 m) and SWIR (30 m) band data into the pixel scale of 90 m to match the ASTER LST data. And calculate the broadband emissivity of ASTER with emissivity of 10–14 obtained from the AST_05 product.
(3) Sub-setting the MODIS image to cover the same geographical region as the ASTER image.
(4) Applying the procedures outlined in Section 3.6 to perform the pixel decomposition of the MODIST LST image into the same pixel scale as the ASTER LST image. Since the ASTER LST image has a pixel scale of 90 m and the MODIS thermal radiance data has resampled the scale to 990 m, there are 11 × 11 sub-pixels in the decomposed LST image for each parent pixel.
(5) Selecting a sample representing the 3 surface patterns from the decomposed LST image and the ASTER LST data obtain from AST_08 data to compute the RMSE for assessment of the accuracy of the approach.
(6) Comparing with other methods for pixel decomposition. In order to assess the efficiency of the approach, we also conduct the decomposition of MODIS LST image using re-sampling technique with linear model and the E-DisTrad method presented in Essa *et al.*, for pixel decomposition of the MODIS LST image [27].

## 4. Results and Discussion

### 4.1. Decomposition of MODIS LST with Its VNIR Data

We applied the procedures of our DSDP approach to MODIS LST images for pixel decomposition with its VNIR data. Initial temperature for each sub-pixel in the decomposed image needs to be firstly estimated so that the weight can be computed for the decomposition. This is done on the basis of the relationships respectively between LST and NDVI for natural terrain, LST and NDBI for urban surface, LST and WCI for water surface. The relationship between LST and remote sensing indices could be obtained from the MODIS LST product and MODIS VNIR auxiliary data as shown in Table 2.

**Figure 6.**
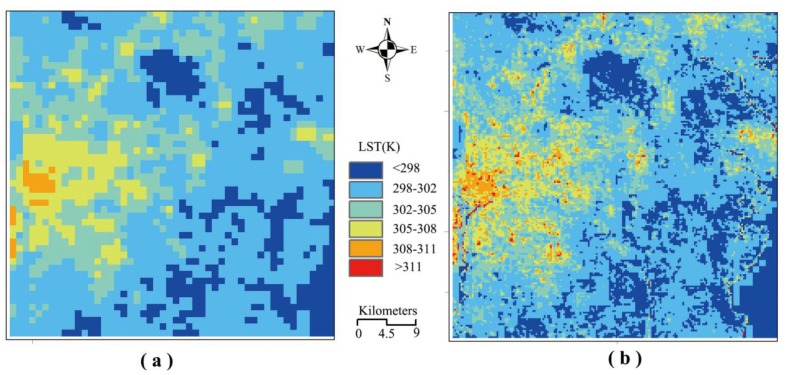
(**a**) MODIS LST image from the MOD11A1 product and (**b**) Decomposed result of the MODIS LST image into the pixel scale of 250 m by DSPD approach.

Using the relationships established in Table 2, we are able to estimate the initial temperature for the decomposed image and to compute the weight for the decomposition. Then we can decompose the experiment MODIS LST image into the pixel scale of 250 m with the procedure in Section 3.6. Figure 6a shows the MODIS LST image from the MOD11A1 product with the pixel scale of 1,000 m and Figure 6b shows the decomposed result of the MODIS LST image into the pixel scale of 250 m by DSPD approach in the Washington DC and Maryland region.

Very similar distribution of the LST can be seen in the two images. Since Figure 6a has a pixel scale of 1000 m while the scale in Figure 6b is 250 m, many more details of LST variation in the Washington DC and Maryland region can be highlighted in Figure 6b than in Figure 6a. The low LST along the Potomac River flowing through Washington DC cannot be clearly identified in the MODIS LST image (Figure 6a), while it is clearly seen in the decomposed LST image (Figure 6b) Many more hot points in Washington DC are shown in the urban area through the decomposed LST image (Figure 6b).

### 4.2. Comparison with the ASTER LST for Validation

**Figure 7.**
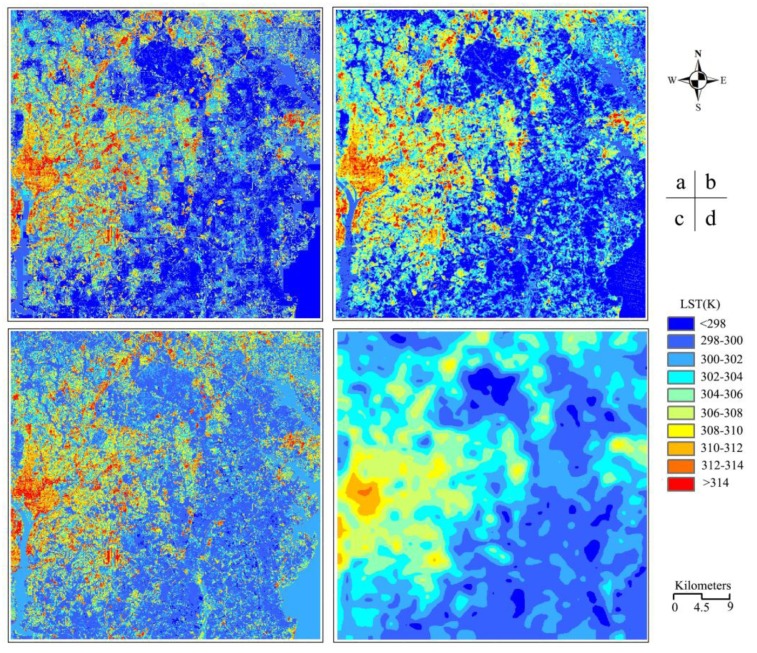
(**a**) Decomposed result of the MODIS LST image into the pixel scale of 90 m by our DSPD approach; (**b**) the corresponding ASTER LST image from the AST_08 product; (**c**) the decomposed results of the MODIS LST by E-DisTrad method (**d**) the LST re-sampling result with cubic convolution model for comparison.

Using the procedure shown in Figure 5, we are able to decompose the entire MODIS LST image into the pixel scale of 90 m. Figure 7a shows the decomposed result of the MODIS LST image. The accuracy of the decomposed LST (Figure 7a) can be assessed through comparison with the simultaneous measurement of LST (Figure 7b) obtain from the ASTER LST product. Very similar distribution of the LST can be seen in the two images (Figure 7a,b), demonstrating that the decomposed LST from the coarse spatial resolution of MODIS LST image is applicable.

In order to demonstrate the applicability of our approach, we also conducted the pixel decomposition of MODIS LST image using the E-DisTrad method and the re-sampled approach used in general image processing. Both E-DisTrad and re-sampled methods are unable to maintain the rule of thermal radiance unchanged after decomposition. Figure 7c,d show the results of these two methods. Obvious difference can be seen between the decomposed LST image by the E-DisTrad method and the re-sampled approach. Much details of LST change can be seen in the result of E-DisTrad method, while the LST distribution tends to be even in the result of re-sampled approach. On the other hand, similar LST distribution can be seen in the results from our approach and the E-DisTrad method because both are based on the relation between LST and remote sensing indices. However, detailed comparison reveals that our decomposed LST image is closer than the decomposed LST image of E-DisTrad method to the ASTER LST image. Table 3 compares the decomposition accuracy of our approach with the E-DisTad and re-sampled methods. As indicated in Table 3, ME and RMSE of our DSPD method is the smallest among the three methods for comparison, which demonstrates that our approach is the best among the three. For the entire image, the ME of our DSPD is −1.29 K, which mean the decomposition result is totally 1.29 K lower than its true result, and the RMSE is 2.7 K, which means that the decomposition accuracy is with an average error of ∼2.7 K in spite of its general applicability. This is mainly attributed to the uncertainty of LST change with NDVI and NDBI used to estimate the initial temperature for the sub-pixels.

Comparison has been done to each of the three typical surface patterns. As seen in Table 3, our DSPD method has the lowest RMSE in all three surface patterns. For natural terrain, the RMSE is 2.1 K for our DSPD, 2.5 K for E-DisTrad and 3.1 K for resampling. For urban surface, the RMSE is 3.6 K for our DSPD, 4.1 K for E-DisTrad and 4.3 K for resampling (Table 3).

The re-sampling method has the highest RMSE for natural terrain and urban surface and the highest RMSE of E-DisTrad method is in water body. Figure 7c indicates that the E-DisTrad method estimates LST to be around 304–306 K for the water body while the ASTER LST is generally <300 K (Figure 7b). Thus it has an overestimate of ∼4 K. Precise comparison has been also conducted to typical areas in the image. Four typical areas were choose for the comparison (Figure 1d). Area A is mainly urban surface while area B is natural terrain. Area C is mainly water body and area D is a mixture of urban surface and natural terrain. Table 3 shows that our DSPD approach has the lowest RMSE among the three methods in these typical areas in comparison of the decomposed LST with the ASTER LST. Our DSPD approach has a RMSE of 2.6 K in area A and 1.8 K in area B, while the RMSE of E-DisTad method is 3.3 K and 2.4 K respectively for areas A and B. The RMSE of re-sampled method is the highest, implying that the method is the worst in decomposing the pixels of coarse LST images into the fine images with high spatial resolution.

### 4.3. Discussion with the Influence of Initial Temperature on DSPD Results

**Figure 8.**
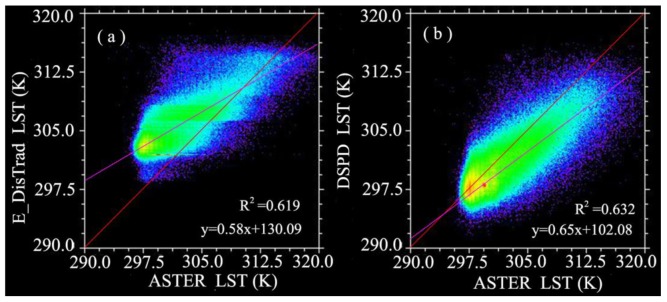
Scatter plot of the decomposed LST images and ASTER LST: (**a**) E_DisTrad result and ASTER LST image; (**b**) DSPD and ASTER LST image.

**Figure 9.**
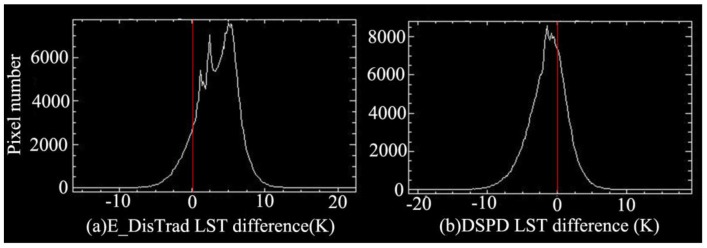
Difference statistic chart of the decomposed LST images and ASTER LST: (**a**) E_DisTrad result and ASTER LST image; (**b**) DSPD and ASTER LST image.

Several studies such as Agam *et al.* [29] and Essa *et al.* [27] used the initial temperature as the final decomposed results because they only based on the relationship between LST and remote sensing indices for the decomposition. However, this is not the best result, because it does not maintain the thermal characteristics of pixel scale to be unchangeable after the decomposition. And Zhu *et al.*, found the relationships of LST and remote sensing indices are variable at different spatial resolution [11]. Meanwhile, the remote sensing indices value ranges are also changeable with spatial resolution. e.g., the maximum NDBI values of MODIS and ASTER are respectively 0.2 and 0.5. So when we used the ASTER NDBI and NDBI-LST relationship of MODIS to obtain an initial decomposed temperature, the initial decomposed temperature may higher than its true value. This higher estimation has been apparently reflected by the scatter plot (Figure 8a) and the difference statistic chart (Figure 9a) between initial decomposed temperature and ASTER LST. While this inaccuracy LST estimation has got some improvement after we used the DSPD method based on the initial decomposed temperature as shown in Figure 8b and Figure 9b.

We respectively made the scatter plot by the result of E_DisTrad and DSPD method with the ASTER LST respectively, the regression line of DSPD is much closer to the red 1:1 reference line with the slop of DSPD regression line (Figure 8b) is 0.65 better than that of E_DisTrad method 0.58 (Figure 8a). The difference statistic chart of the decomposed LST and ASTER LST (Figure 9b) also indicated a satisfactory estimation with our DSPD method than E_DisTrad method.

Since the E-DisTrad method may generate some error in the LST disaggregation process, we need to make some hypothesis to evaluate how much the error in the initial temperature estimated by E-DisTrad method would finally influence the DSPD result. For a MODIS LST pixel could decompose to 16 sub-pixels with its VNIR data, it is difficult to evaluate error of each sub-pixel could influence the final result. So we make an extreme hypothesis, the 16 sub-pixels composed by 15 vegetation sub-pixels (low LST) and 1 urban sub-pixel (high LST), and the initial temperature error of vegetation and urban sub-pixels is in the range of ±3 K. Under this hypothesis, the MODIS LST and the parameters of its sub-pixels were given in Table 4.

**Figure 10.**
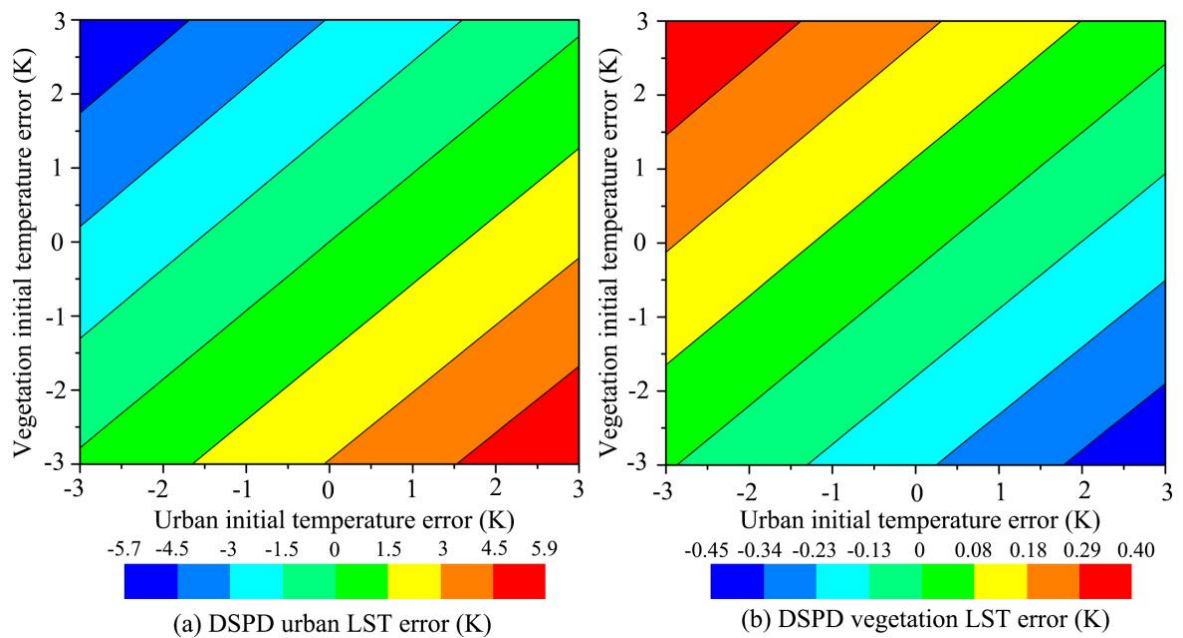
DSPD LST error of (**a**) urban sub-pixel and (**b**) vegetation sub-pixels under different initial temperature estimation error under hypothesis situation.

**Figure 11.**
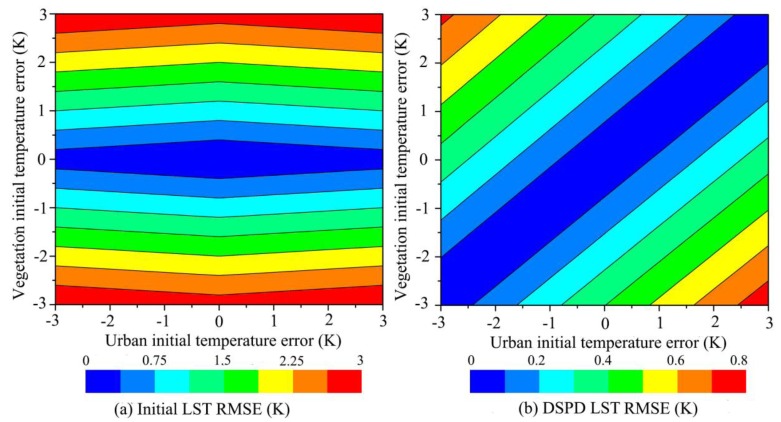
RMSE of total sub-pixels under hypothesis situation by (**a**) E-DisTrad and (**b**) DSPD.

The object of DSPD method is to maintain the thermal radiance to be unchangeable after the decomposition. If the 15 vegetation sub-pixels among total 16 sub-pixels have the same estimated initial temperature error, the thermal radiance conducted by this error will intentionally reflect on the other one urban sub-pixel. As shown in Figure 10, we give the DSPD LST error of urban sub-pixel and vegetation sub-pixels under different initial temperature estimation error. An interesting phenomena will be found, if the vegetation initial temperature error and the urban initial temperature error increase or decrease together, the DSPD LST result of urban and vegetation will both have little error with the green color, because the weight for the sub-pixels change little under this situation. Meanwhile, if the vegetation initial temperature error and the urban initial temperature error increase or decrease inversely, the DSPD LST result of urban and vegetation will have high error with the blue or red color. This means if the 15 vegetation sub-pixels initial temperature are higher estimated and urban sub-pixel initial temperature are lower estimated, the DSPD urban sub-pixel LST will be much lower than its true LST (the blue region of Figure 10a) while the DSPD vegetation sub-pixel LST will be little higher than its true LST (the red region of Figure 10b). Under the maximum situation, the vegetation sub-pixels initial temperature have 3 K higher estimation and the urban sub-pixel initial temperature has 3 K higher estimation, the DSPD urban sub-pixel LST will be 5.7 K lower than its true LST while the DSPD vegetation sub-pixels LST will only be 0.4 K higher than its true LST. And if the 15 vegetation sub-pixels initial temperature are lower estimated and urban sub-pixel initial temperature are higher estimated, the DSPD urban sub-pixel LST will be much higher than its true LST (the red region of Figure 10a) while the DSPD vegetation sub-pixel LST will be little lower than its true LST (the blue region of Figure 10b). Although the DSPD may obtain a large error under the maximum situation in the urban sub-pixel, the error RMSE of total 16 sub-pixels with DSPD is 0.8 K much less than the RMSE of initial temperature RMSE of 3 K (Figure 11), so the error RMSE of DSPD is totally better than the initial temperature.

### 4.4. Applying the Approach to Another Case

**Figure 12.**
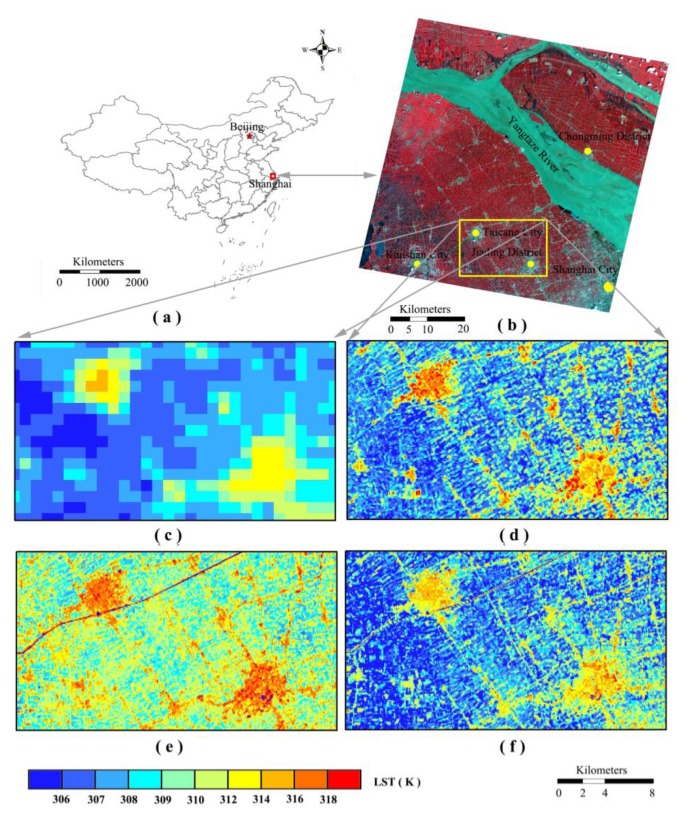
Applying the DSDP approach to the MODIS and ASTER LST image in east China, (**a**) the location of the data used in Shanghai; (**b**) the ASTER image, RGB: 3N21; (**c**) the MODIS LST subset covering the Shanghai region; (**d**) the ASTER LST subset covering the same region; (**e**) decomposed results of the MODIS LST into the pixel scale of 90 m by E-DisTrad method; (**f**) decomposed result of the MODIS LST image into the pixel scale of 90 m by our DSPD approach.

We applied the procedures of our DSDP approach to another site of Shanghai in east of China (Figure 12a). With rapid development of economy, urbanization is very obvious in the region, especially Shanghai City, the LST product with high spatial resolution is the essential data for urbanization study. As shown in Figure 12. The ASTER and MODIS LST in Shanghai area were acquired at 02:47:07 Greenwich time (10:47:7 local time) on 1 August 2000. Figure 12c shows the subset of the MODIS LST image and Figure 12d is the subset of the ASTER LST image covers Shanghai region, the Taicang city and Jiading district. The decomposed results of E_DisTrad (Figure 12e) and our DSPD (Figure 12f) all could reflect the spatial distribution of high LST in the urban building area and low LST in the vegetation and water area. The decomposed LST result of E_DisTrad has an apparently high estimation compared with the ASTER LST data. The LST in vegetation area with about 306 K is highly estimated to 310 K, and the hot points in the urban area are much more. This high estimation result has been effectively decreased after our DSPD method. The decomposed LST result of DSPD in the vegetation area is lower than the E_DisTrad result and much similar with the observed ASTER LST. We would find the decomposed LST result of DSPD in the urban area of Taicang city and Jiading district is about 2 K lower than ASTER LST. This is mainly attributed to the discrepancy between the ASTER and MODIS LST product in this area [45]. Therefore our DSPD method provides an alternative approach to improve the spatial resolution of thermal remote sensing system such as MODIS with coarse pixel scale.

## 5. Conclusions

An efficient approach has been proposed in the study to increase the spatial resolution of MODIS LST image. The theoretical basis of the approach is that the change of LST is with accordance with characteristics of surface patterns. Therefore, using a scheme to classify the image pixels into three surface patterns and establish the relationship between LST and NDVI or NDBI, we are able to decompose the pixel scale of the MODIS LST image into the same pixel scale of MODIS VNIR bands which are used as auxiliary data for the decomposition. Since the pixel scale of MODIS VNIR bands is 250 m, we are able to increase the spatial resolution of the MODIS LST image into 3 times, *i.e.*, to decompose the pixel scale of the MODIS LST image from its original 1000 m into 250 m in the resulted LST image. The unique feature of this pixel decomposition approach is to maintain the thermal radiance of parent pixels in the MODIS LST image unchanged after they are decomposed into the sub-pixels in the resulted image. There are two important steps in the decomposition: initial temperature estimation for the sub-pixels in the resulted LST image and final temperature determination through weighting approach to maintain the thermal radiance unchanged after decomposition. Therefore it is termed the double-step pixel decomposition (DSPD).

A series of procedures have been developed for the pixel decomposition approach. These include re-sampling the MODIS VNIR data, classification of the image pixels into three surface patterns, establishment of the relationship between LST and NDVI or NDBI or WCI for the three surface patterns, computation of weight for each sub-pixel, and determination of final temperature for the sub-pixels. An experiment was conducted in the study to validate the applicability of the approach. Because ASTER and MODIS are on board the same platform for Earth observation, the ASTER LST which has much higher spatial resolution than the MODIS LST image was used as the true LST measurement to compare with the decomposed LST from the MODIS LST image. We compared the DSPD approach with the E-DisTrad method and re-sampling method.

The comparison results indicate our DSPD approach is the best among the three. RMSE of our DSPD is 2.7 K for the entire image used for the experiment. Re-sampling method has the highest RMSE, implying that it is the worst for pixel decomposition. This is mainly because the method does not consider the effect of surface patterns on LST variation in its decomposition, while the DSPD and E-DisTrad methods are based on this effect for decomposition. The obvious difference of our DSPD from the E-DisTrad is that the thermal radiance remains unchanged after decomposition while E-DisTrad does not emphases this principle.

In spite of general applicability, the decomposition accuracy is not as high as expected. For our DSPD, its RMSE indicates that the decomposition is with an average error of ∼2.7 K. This is mainly attributed to the uncertainty of LST change with NDVI and NDBI used to estimate the initial temperature for the sub-pixels. Decomposition accuracy is different in different surface patterns. The lowest RMSE can be seen in the water body, which is followed by natural terrain. The urban region has the highest RMSE for the decomposition. This is due to the fact that LST has the lowest fluctuation in water bodies and the greatest variation in urban region.

The DSPD approach has been applied to MODIS LST images in east USA and east China for pixel decomposition to increase their spatial resolution. Since the MODIS NVIR band data were used as assistance for the decomposition, the resulted LST images from the decomposition efficiently increase the spatial resolution in both cases. The pixel scale of MODIS LST images were 1000 m while the scale of the decomposed LST images is 250 m, implying that the spatial resolution of the LST image has increased by three times after decomposition. The obvious increase in spatial resolution remarkably uplifts the capability of the LST images in applying to the studies requiring high spatial resolution of LST distribution to highlight the tiny variation of thermal flux over ground surface. Much more details of LST variation can be clearly identified in the decomposed LST image than in the original MODIS LST. Therefore it can concluded that the DSPD approach provide an alternative opportunity to improve the spatial resolution of MODIS LST images hence expand the applicability of the images in the real world.

## Figures and Tables

**Table 1.** Coefficient of thermal radiance and temperature of the blackbody at different wavelength ranges.

**Wavelength (μm)**	***K*_1_ (W·m^−2^)**	***K*_2_ (K)**
8–13.5	17,890	1411
10.78–11.28	1321	1339

**Table 2.** LST and remote sensing indices relationship with the MODIS LST and VNIR data

**Landcover**	**LST-RS Indices**	***R*^2^**
Nature surface	LST*n* = 310.085 − 18.654NDVI	0.7555
Urban	LST*u* = 304.884 + 35.212NDBI	0.7218
Water	LST*w* = 296.258 + 3.406WCI	0.7603

**Table 3.** Comparison of LST error statistics indices for different decomposition methods.

**Cases**	**DSDP Method**			**E-DisTrad Method**			**Re-Sampled Method**		

	**ME**	**STD**	**RMSE**	**ME**	**STD**	**RMSE**	**ME**	**STD**	**RMSE**
Entire image	−1.29	2.73	2.72	3.47	2.79	3.32	−1.50	3.49	3.58
Natural terrain	−0.96	2.94	2.12	6.13	3.34	2.48	−0.82	3.20	3.09
Urban surface	−1.94	2.54	3.59	−1.27	2.35	4.15	−2.86	4.22	4.34
Water bodies	−1.08	1.12	0.31	1.32	0.94	4.04	−1.12	1.42	2.19
Area A	0.15	2.19	2.61	−1.15	3.02	3.25	−1.52	2.48	3.98
Area B	−0.58	1.71	1.78	6.02	1.26	2.43	−1.02	1.03	2.81
Area C	0.23	0.27	0.22	2.12	0.84	1.14	0.33	0.32	1.32
Area D	−1.25	3.22	2.81	2.91	3.23	3.85	−1.37	2.22	4.68

Note: Geographic location of the areas A, B, C, and D is shown in Figure 1d.

**Table 4.** Parameters of the MODIS LST sub-pixels.

**Vegetation BBE**	**Urban BBE**	**Vegetation (K)**	**Urban (K)**	**MODIS Thermal Radiance (W·m^−2^)**
0.96	0.92	300	312	158.5876
